# Symmetry Breaking of B_2_N^(−, 0, +)^: An Aspect of the Electric Potential and Atomic Charges

**DOI:** 10.3390/molecules201219769

**Published:** 2015-12-03

**Authors:** Majid Monajjemi, Samira Bagheri, Matin S. Moosavi, Nahid Moradiyeh, Mina Zakeri, Naime Attarikhasraghi, Nastaran Saghayimarouf, Ghorban Niyatzadeh, Marzie Shekarkhand, Mohammad S. Khalilimofrad, Hashem Ahmadin, Maryam Ahadi

**Affiliations:** Department of Chemistry, Science and Research Branch, Islamic Azad University, Tehran 801, Iran; s.bagheri.sm@gmail.com (S.B.); majid_kuala@yahoo.com (M.S.M.); moradiyeh.nahid@gmail.com (N.M.); zakeri.mina@srbiau.ac.ir (M.Z.); naimeattari.kh@gmail.com (N.A.); n.saghayi@gmail.com (N.S.); m_monajjemi@yahoo.com (G.N.); marzieh.shekarkhand@yahoo.com (M.S.); Mohammadsajjad_khalili@yahoo.com (M.S.K.); araz.a1388@gmail.com (H.A.); maryam.ahadi1986@gmail.com (M.A.)

**Keywords:** symmetry breaking, electric potential, atomic charges, Boron Nitride compounds, h-BN sheet

## Abstract

In this study, the three forms of B_2_N^(−, 0, +)^—radical, anion and cation—have been compared in terms of electric potential and atomic charges, ESP, rather than the well-known cut of the potential energy surface (PES). We have realized that the double minimum of the BNB radical is related to the lack of the correct permutational symmetry of the wave function and charge distribution. The symmetry breaking (SB) for B_2_N^(0, +)^ exhibits energy barrier in the region of (5–150) cm^−1^. The SB barrier goes through a dynamic change with no centrosymmetric form which depends on the wave function or charge distribution. In spite of ${\tilde{A}}^{2}\Sigma_{g}^{+}$ exited state, the${\tilde{\;B}}^{2}\prod_{g}$ excited configuration contributes to the ground state (${\tilde{\;B}}^{2}\prod_{g}-{\tilde{X}}^{2}\Sigma_{u}^{+})$ for forming radicals. The SB did not occur for the anion form (B_2_N^(−)^) in any electrostatic potential and charges distribution. Finally, we have modified the Columbic term of the Schrödinger equation to define the parameters “αα' and ββ*'*” in order to investigate the SBs subject.

## 1. Introduction

Linear triatomic structures, particularly those of the X-Y-X type are one of the simplest molecular systems with “high” symmetry in which symmetry breaking (SB) may occur. The point group of the BNB in the linear symmetric X-Y-X species is “*D*_∞*h*_” whereas its asymmetrically distorted form has the symmetry of the *C*_∞*v*_ group. Tri-atomic B_2_N^(−, 0, +)^ molecules have been subsequently studied using a variety of calculations and spectroscopic methods. It is a deep challenge to measure the real or artifactual SB effects due to its capability to a display a pseudo second-order Jahn–Teller effect, which results in a structure with unequal BN bond lengths [1,2,3,4,5,6,7,8,9,10,11,12,13,14].

Obviously, the adiabatic system of the Born–Oppenheimer approximation breaks down when the electronic states are degenerate, so the SB can be associated with degeneracy. SB may result from first-order Jahn–Teller effects or Renner–Teller effects for degenerate electronic states, while non-degenerate states result from interactions between different states,* i.e.*, the so-called pseudo Jahn–Teller (SOJT) effects.

The first important investigation for BNB was done by Martin* et al. *in 1989. Based on an UHF/6-31G* geometry optimization, they predicted that the B_2_N has a symmetric linear regulation in its ground state (${\tilde{X}}^{2}\Sigma_{u}^{+})$) with an unusually low bending frequency (73 cm^−1^) [1].

They noted that the full valence CASSCF wave function for the ${\tilde{X}}^{2}\Sigma_{u}^{+}$ state of linear B–N^(0)^–B is unstable with respect to symmetry lowering,* i.e.*, the *C*_∞*v*_ structure (*r*_1(*BN*)_ ≠ *r*_2(*BN*)_) yields a lower total energy than the *D*_∞*h*_ symmetric structure (*r*_1(*BN*)_ = *r*_2(*BN*)_) [10].

In 1992, Knight* et al. *found two minimum structures,* i.e.*, the linear B_2_N^(0)^ and cyclic B_2_N [4]. The UHF/6-31G* level of theory predicts the cyclic ^2^B_2_ state to be at the global minimum, while the correlated methods predict the ${\tilde{X}}^{2}\Sigma_{\left(u\right)}^{+}$ state of linear B–N–B to be at the global minimum and the cyclic ^2^B_2_ state to be at the local minimum.

There are two conceivable valence bond structures for open-shell system of the BNB radical in the ground state. The first one is the localized form where the symmetry is *C*_∞*v*_{^2^Σ^+^:1σ^2^, 2σ^2^, 3σ^2^, 4σ^2^, 5σ^2^, 1π^4^, 6σ^2^, 7σ^1^}, and the second is the resonance form where the symmetry is *D*_∞*h*_ {${(}^{2}\Sigma_{u}^{+}):1\sigma_{g}^{2},1\sigma_{u}^{2},2\sigma_{g}^{2},3\sigma_{g}^{2},2\sigma_{u}^{2},1\pi_{u}^{4},4\sigma_{g}^{2},3\sigma_{u}^{1}$}. Obviously describing the electronic structure of the open-shell system (B_2_N^(0)^) is much more difficult than the closed-shell system of B_2_N^(−, +)^ and in many cases, the description of an open-shell system is challenging in computational quantum chemistry calculations. This is primarily due to the presence of static correlation effects (requiring a multireference-type description) [13,14].

In general, researchers agree that BNB is linear in its ground electronic state of (${\tilde{X}}^{1}\Sigma_{g}^{+}$) and (${\tilde{X\;}}^{2}\Sigma_{u}^{+})$ for anionic and neutral forms, respectively [2,13].The ground state of the B*N*^(0)^B has been examined via the developed reduced multireference coupled cluster method with singles and doubles that is perturbatively corrected for triples [RMR CCSD (T)] using the correlation consistent basis sets (cc-pV*D*Z, cc-pV*T*Z and cc-pV*Q*Z) by J. Paldus [9]. They showed that the ground state has an asymmetric structure *C*_∞*v*_ with two BN bonds of unequal length.

In an experimental study, the state of ${\tilde{A\;}}^{2}\Sigma_{g}^{+}(B_{2}N^{-})$ was observed in photoelectron spectroscopic studies (PES) by Asmis *et al.*, placing the zero-point level of the 6330 ± 40 cm^−1^ above the ground state of the ${\tilde{X}}^{2}\Sigma_{\left(u\right)}^{+}$ [2]. He showed that the observed signal in the 355 and 266 nm photoelectron spectra of *B*_2_N^−^ has been indicated to a photodetachment from the anion ground state ${\tilde{(X}}^{1}\Sigma_{g}^{+})$ to the ground and lowest excited states of neutral B_2_N* i.e.*, ${\tilde{X}}^{2}\Sigma_{u}^{+}\;$ and $\;{\tilde{A}}^{2}\Sigma_{g}^{+}\;$ with a linear symmetry and is assigned to the ${\tilde{X}}^{1}\Sigma_{g}^{+}\;\to {\tilde{X}}^{2}\Sigma_{u}^{+}+\;e^{-}$ and ${\tilde{X}}^{1}\Sigma_{g}^{+}\;\to {\tilde{A}}^{\;2}\Sigma_{g}^{+}+\;e^{-}$ transitions.

Electron spin resonance (ESR) experiments indicate the unpaired electron between the two B atoms implying symmetric geometry [4]. However, this result cannot exclude the presence of a small barrier to asymmetry in point view of the time scales involved. A further, matrix infrared study [7] indicated that the ground state of BNB is cyclic, whereas a more recent photoelectron spectroscopic study of B_2_N^(−)^ anion [2] confirmed a linear symmetric geometry. Furthermore, the infrared absorptions were also observed in the cryogenic argon matrix near 6000 cm^−1^ of the electronic band system, due to the ${\tilde{A\;}}^{2}\Sigma_{g}^{+}-{\tilde{X\;}}^{2}\Sigma_{u}^{+}$ [7].

Recently, the compounds of BNB, especially their electronic structures have been considered [15,16,17,18,19]. In 2009, a series of multi-reference approaches based on the SA-CASSCF wave function,* i.e.*, CASPT2, MRCI, and MRAQCC, have been employed by Boggs and coworkers to investigate the SB in the ground state ${\tilde{X}}^{2}\Sigma_{\left(u\right)}^{+}$ of the triatomic B_2_N^(0)^ radical [20]. Their results show that B_2_N in its ground state has a linear non-centrosymmetric structure with two equivalent global minima of the adiabatic potential energy surface, including two oppositely directed dipole moments, respectively. They accepted that the PJT effect involving vibronic interaction with the first excited state ${\tilde{A}}^{2}\Sigma_{\left(g\right)}^{+}$ via the asymmetric stretching vibrations is the major reason for the double-minimum. On the other hand, the large-scale multi-reference configuration interaction calculations, CASSCF+1+2 predicted an asymmetric configuration, while the SACASSCF+1+2 predicted a symmetric *D*_∞*h*_ ground state [21].

In another study in 2010, Stanton has discussed an unusually large non-adiabatic error in the BNB molecule as well as non-adiabatic corrections to energy level [22]. He illustrated those non-adiabatic corrections to energy levels should fall out only when the affected vibrational frequency is large enough to be of comparable magnitude to the energy gap. In other words, non-adiabatic corrections should be given as much weight as issues such as high-level electron correlation, relativistic corrections, *etc.*

In other words, calculations using larger and larger basis sets, and more and more advanced methods of electron correlation, are doomed to approach the “wrong” limit for the vibronic levels of BNB if the Born-Oppenheimer approximation is applied, so the electrostatic potential charges which are based on wave functions of the BNB systems and are related to quantum mechanics phenomena can be used as suitable tools through the significant approach reported in this work.

In 2013, Kalemos [21] tried to approach the SB problem by using high level multi-reference variation and full configuration interaction methods. He indicated that the (SB) problem is related to the lack of the “correct” permutation symmetry of the wave-functions adopted to attack the problem and is by no means a real effect. Furthermore, he indicated the wave-function which is not invariant under all symmetry operations of the point group should be symmetrically broken (SB). SB (in classical mechanics) occurs when a stable minimum undergoes splits into two stable minima. He checked the MRCI results by FCI (9e^−^)/[3*s*2*p*] calculations and found no trace of SB in qualitative disagreement with all previous theoretical investigations that predicted a barrier to a centro-symmetric structure either of 20 cm^−1^ (based on MRCI methods of Boggs) or of 100–160 cm^−1^ (based on CC methods) [9,22,23].

**Scheme 1:**
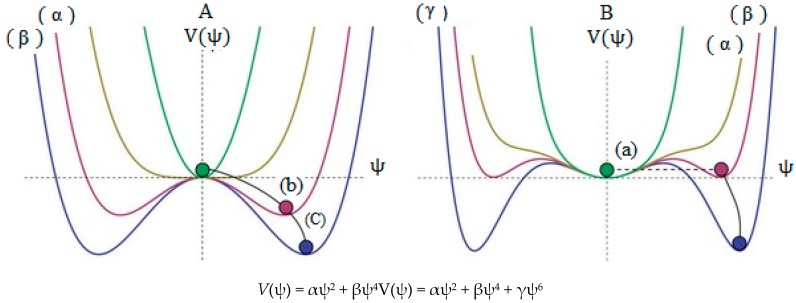
A simplified of spontaneous symmetry breaking, (a) indicates high energy level which the ball settles in the center, and the result is symmetrical; (b) and (c) are lower in energy levels and the overall “rules” remain symmetrical, however, as the potential comes into effect, the “local symmetry” is inevitably broken since eventually the ball must roll one way (at random) and not another; (**A**) is a non-linear combination of two wave function and (**B**) is a non-linear combination of three wave function.

In this study, we compare three forms of B_2_N^(−, 0, +)^ (anion, radical and cation) in terms of electrostatic potential charges “ESP” rather than the cut of the potential energy surface (ESP is changed using the trial wave functions). For a charged system with charge *Q*, the density |$\psi \left(x\right){|}^{2}$ multiplied by the atomic charge yields the charge density |$Q\left(x\right){|}^{2}$. Large points for fitting of various situations have been used to calculate the atomic charges and electrostatic potential of the systems. As a result, the possibility of an asymmetric ground state may not be eliminated or it seems that an asymmetric geometry is a rather comfortable situation; the double minimum nature of BNB is related to the lack of the correct permutation symmetry of the wave functions (Scheme 1).

The small barrier energy including the two equal minima in some parts of B_2_N^(−, 0, +)^ systems have been estimated using symmetrical linear combination of wave functions (SLC-WFs). These barriers are dependent on the charge distribution, SLC-WFs and correlation effects between |α〉 or |β〉 levels which are not global minima.

There are 16, 17, and 18 electrons in B_2_N^(−, 0, +)^ systems. Therefore, based on Walsh prediction [24], it appears that the linear *p*-block molecule holds up quite well for all B_2_N^(−, 0, +)^ ions and the radical. This work has focused on a spontaneous symmetry breaking (SSB) [25,26,27] for B_2_N^(−, 0, +)^ systems in view of ESP.

## 2. Wave Functions and Symmetry Breaking

SB is generally applied to two phenomena—the first is the failure of the electronic wave function to transform as an irreducible representation of the molecular point group and the second is the preference of the nuclear framework for lower-symmetry geometry [28]. The failure of the electronic wave function is purely artifactual in that the exact wave function necessarily obeys the symmetry properties of the molecular point group and the symmetry-contaminated wave functions was described by Lowdin as the symmetry dilemma [29]. In such cases, a broken-symmetry wave function may yield a solution with lower energy than the symmetry-adapted one.

On the other hand, in the absence of external fields, the motion of nuclei is governed by a function of nuclear coordinates which is a sum of the Coulomb repulsion between nuclei and the electronic effective potential terms, resulting from the Born Oppenheimer approximation and wave function, so the meaning of the wave function can be interpreted by the charge density distributions of electrons in a quantum system [30].

It is obvious that every wave function must be satisfactory before being submitted to the Schrödinger equation; however, it has been shown by Wigner that exact electronic wave functions satisfy the Pauli Exclusion Principle [31]. The physical root of charge density is one of the main questions in electronic wave function origin and the key to unveiling the meaning of the wave function is to find the physical root of the charge density [32,33,34,35].

Many questions have been raised from interpretation of the wave function in the theory which is dependent on SB problem such as regarding the wave function as a field similar to electromagnetic field [36], an active information field [37], and a field carrying energy and momentum [38].

In a time independent system using Born-Oppenheimer approximation, the Hamiltonian of boron and nitrogen nuclei are sum of the kinetic energy term and effective potentials (Equation (1)):

(1) $${\hat{H}}_{n}=\overset{3}{\underset{j=1}{\sum }}-\frac{\hbar^{2}}{2M_{j}}\Delta_{j}+V\left({\vec{R}}_{B_{1}},{\vec{R}}_{N},{\vec{R}}_{B_{2}}\right)$$

The B_2_N^(−, 0, +)^ forms will be stable by minimization of “*V*”. So, it depends on sum of Coulomb repulsions between nuclei and the electronic effective potential:

(2) $${\hat{V({\hat{R}}_{B},{\hat{R}}_{N},R}}_{B})=\overset{2}{\underset{j=1}{\sum }}\overset{3}{\underset{l=j+1}{\sum }}\frac{q_{j}q_{l}}{4{\pi \varepsilon }_{0}|{\vec{R}}_{j}-{\vec{R}}_{l}|}\;+\text{E}\left({\vec{R}}_{B_{1}},{\vec{R}}_{N},{\vec{\;R}}_{B_{2}}\right)$$

The dynamic representation was first postulated by Wigner in 1930 with the justification that its action does not change the relative distances between nuclei and is therefore the symmetry of the dynamical problem [31]. Damljanovic modified the Wigner postulate in another way for dynamical representation, while for every molecule; there exists at least one normal mode that belongs to the totally symmetric irreducible representation of the point group of that molecule [34]. In this approach, the problem of finding minima of *V *becomes another case of the spontaneous symmetry breaking phenomenon and it allows finding approximate relations between bond lengths in a molecule and its vibrational frequencies. The Schrödinger equation for electrons moving in the field generated by nuclei in a molecule subjected to no external fields can be written as follows:

(3) $$[\overset{N_{e}}{\underset{j=1}{\sum }}-\frac{\hbar^{2}}{2m_{e}}.\left(\frac{\partial }{\partial {\vec{r}}_{j}^{a}}{)}^{2}-\overset{N_{e}}{\underset{j=1}{\sum }}\sum_{l=1}^{N}\frac{eq_{\left(l\right)}}{4{\pi \varepsilon }_{0}|{\vec{r}}_{j}^{a}-{\vec{R}}_{j}^{a}|}+\;\overset{N_{e}-1}{\underset{j=1}{\sum }}\sum_{l=j+1}^{N_{e}}\frac{e^{2}}{4{\pi \varepsilon }_{0}|{\vec{r}}_{j}^{a}-{\vec{r}}_{l}^{a}|}\right]\psi \left({\vec{r}}^{a},{\vec{R}}^{a}\right)=\text{E }\psi \left({\vec{r}}^{a},{\vec{R}}^{a}\right)$$

where ${\vec{r}}^{a}={\left({\vec{r}}_{1}^{a},\ldots \;{\vec{r}}_{N_{e}}^{a}\right)}^{T}$ and ${\vec{R}}^{a}=\;{\left({\vec{R}}_{1}^{a},\ldots \;{\vec{R}}_{N_{e}}^{a}\right)}^{T}$ and *T *is the transposition vector in the 3*N*-dimesional real vector space. In this equation, the first term in the left is kinetic energy of the electrons, the second term is Coulomb attraction between electrons and nuclei and the third term is Coulomb repulsion between electrons and coordinates of nuclei that are the parameters on which the eigenvalue *E *is dependent “E = E(${\vec{R}}^{a}$)”. Damljanovic has discussed that any two configurations of nuclei can be obtained from each other by rotation, translation or a permutation of one configuration as a whole correspond to the same value of *V* or *E * [39]*.*

Since Coulomb repulsion between nuclei diverges at origin, the function “V” cannot be expanded by the use of Taylor formula around that point. On the other hand, “E” is finite at the point R = 0. It is equal to the electronic energy of an atom (called united atom) having charge of the nucleus equal to sum of charges of all nuclei in the molecule under investigation. Moreover, stable configurations of relatively simple molecules are confined in space in the small volume around an origin.

We have modified our systems based on definition of “V” in order to find minima as follows:

(4) $$V=\alpha [{\left({\vec{R}}_{N}-{\vec{R}}_{B_{1}}\right)}^{2}]+\beta [\left({\vec{R}}_{N}-{\vec{R}}_{B_{2}}{)}^{2}\right]+\gamma [{\left({\vec{R}}_{B_{1}}-{\vec{R}}_{B_{2}}\right)}^{2}]+\frac{1}{4{\pi \varepsilon }_{0}}[\frac{\alpha^{\prime }q_{N}q_{B_{1}}}{\left|{\vec{R}}_{N}-{\vec{R}}_{B_{1}}\right|}+\frac{\beta^{\prime }q_{N}q_{B_{2}}}{\left|{\vec{R}}_{N}-{\vec{R}}_{B_{2}}\right|}+\frac{\gamma^{\prime }q_{B_{1}}q_{B_{1}}}{\left|{\vec{R}}_{B_{1}}-{\vec{R}}_{B_{2}}\right|}]$$

where ${\vec{R}}_{N},{\vec{R}}_{B_{1}},\;{\vec{R}}_{B_{2}}$ and charges *q_N_*, $q_{B_{1}},q_{B_{2}}$ are bound distances and charge distribution of B_2_N^(−, 0, +)^ and ${\vec{\lambda }}_{1}$, ${\vec{\lambda }}_{2}$ and ${\vec{\lambda }}_{3}$ are defined as:

(5) $${\vec{\lambda }}_{1}={\vec{R}}_{N}-{\vec{R}}_{B_{1}},\;{\vec{\lambda }}_{2}={\vec{R}}_{N}-{\vec{R}}_{B_{2}}\text{ and }{\vec{\lambda }}_{3}={\vec{R}}_{B_{1}}+{\vec{R}}_{B_{2}}+{\vec{R}}_{N}\text{ and V }=\alpha {{\vec{\lambda }}_{1}}^{2}+\beta {{\vec{\lambda }}_{2}}^{2}+\gamma {\left({\vec{\lambda }}_{1}-{\vec{\lambda }}_{2}\right)}^{2}+\frac{1}{4\pi \varepsilon_{0}}[\frac{\alpha^{\prime }q_{N}q_{B_{1}}}{\left|{\vec{\lambda }}_{1}\right|}+\frac{\beta^{\prime }q_{N}q_{B_{2}}}{\left|{\vec{\lambda }}_{2}\right|}+\frac{\gamma^{\prime }q_{B_{1}}q_{B_{1}}}{\left|{\vec{\lambda }}_{1}-{\vec{\lambda }}_{2}\right|}]$$

${\vec{\lambda }}_{1}={\vec{\lambda }}_{2}={\vec{\lambda }}_{0}$, in the center of mass of BNB molecules, while:

(6) $$V(\vec{\lambda })=2\;\alpha |{\vec{\lambda }}^{2}|+\frac{2}{4{\pi \varepsilon }_{0}}\frac{\alpha^{\prime }q_{N}q_{B}}{\left|\vec{\lambda }\right|}\text{ and in minimum}:\;4\alpha |{\vec{\lambda }}_{0}|=\frac{2}{4{\pi \varepsilon }_{0}}\frac{\alpha^{\prime }q_{N}q_{B}}{\left|{\vec{\lambda }}_{0}\right|}\to q_{N}q_{B}=8{\pi \varepsilon }_{0}\alpha \alpha^{\prime }|{{\vec{\lambda }}_{0}}^{3}|$$

Depending on the radical, cation and anion forms of BNB, *q_N_* × *q_B_* multiplication can be positive or negative and consequently, ⎢${\vec{\lambda }}_{0}⎢=\sqrt[{3}]{\frac{q_{N}^{\left(0\right)}q_{B}^{\left(0\right)}}{8{\pi \varepsilon }_{0}\alpha \alpha^{\prime }}}$ would be either positive or negative.

In our calculations, the charges for nitrogen were changed between +0.75 < *q_N_* <+0.87 (for the anion form) and in equilibrium the charges of the nitrogen and the two borons are *q_N_* = 0.868802 and $q_{B_{1}}=q_{B_{2}}=q_{B}=-0.934401$. Therefore, the ${\vec{\lambda }}_{0}=$ 1.329 and αα' = ββ*'* = 1.506 can be yielded in the center.

${\vec{\lambda }}_{1}\neq {\vec{\lambda }}_{2}$,${{\vec{\;\lambda }}_{1}}^{\left(0\right)}$ and ${{\vec{\lambda }}_{2}}^{\left(0\right)}$ denote a stable configuration, and V can be expanded around this point up to the second order as Equation (10):

(7) $$\frac{1}{\left|\vec{\lambda }+\delta \vec{\lambda }\right|}\approx \frac{1}{\left|\vec{\lambda }\right|}-\frac{{\vec{\lambda }}^{\left(0\right)}\vec{\lambda }}{\left|{\vec{\lambda }}^{3}\right|}-\frac{1}{2}\frac{{\left(\delta \vec{\lambda }\right)}^{2}}{\left|{\vec{\lambda }}^{3}\right|}+\;\frac{3}{2}\frac{{\left({\vec{\lambda }}^{\left(0\right)}\delta \vec{\lambda }\right)}^{2}}{\left|{\vec{\lambda }}^{5}\right|}$$

(8) $$\{\left(2\alpha +2\beta +2\gamma -\frac{\alpha^{\prime }q_{N}q_{B_{1}}}{4{\pi \varepsilon }_{0}|{{\vec{\lambda }}_{1}^{\left(0\right)}}^{3}|}-\frac{\gamma^{\prime }q_{B_{1}}q_{B_{2}}}{4{\pi \varepsilon }_{0}|{\left({\vec{\lambda }}_{1}^{\left(0\right)}-{\vec{\lambda }}_{2}^{\left(0\right)}\right)}^{3}|}\right)\}({\vec{\lambda }}_{1}^{\left(0\right)})+\;\left\{\left(-2\gamma -\frac{\gamma^{\prime }q_{B_{1}}q_{B_{2}}}{4{\pi \varepsilon }_{0}|{\left({\vec{\lambda }}_{1}^{\left(0\right)}-{\vec{\lambda }}_{2}^{\left(0\right)}\right)}^{3}|}\right)\right\}({\vec{\lambda }}_{2}^{\left(0\right)})=0$$

(9) $$\{\left(-2\gamma -\frac{\gamma^{\prime }q_{B_{1}}q_{B_{2}}}{4{\pi \varepsilon }_{0}|{\left({\vec{\lambda }}_{1}^{\left(0\right)}-{\vec{\lambda }}_{2}^{\left(0\right)}\right)}^{3}|}\right)\}({\vec{\lambda }}_{1}^{\left(0\right)})+\;\left\{\left(2\alpha +2\beta +2\gamma -\frac{\beta^{\prime }q_{N}q_{B_{2}}}{4{\pi \varepsilon }_{0}|{{\vec{\lambda }}_{2}^{\left(0\right)}}^{3}|}-\frac{\gamma^{\prime }q_{B_{1}}q_{B_{2}}}{4{\pi \varepsilon }_{0}|{\left({\vec{\lambda }}_{1}^{\left(0\right)}-{\vec{\lambda }}_{2}^{\left(0\right)}\right)}^{3}|}\right)\right\}({\vec{\lambda }}_{2}^{\left(0\right)})=0$$

so, the distance between B and N is equal to $|{\vec{\lambda }}_{BN}|=\sqrt[{3}]{\frac{q_{N}^{\left(n\right)}q_{B}^{\left(n\right)}}{8{\pi \varepsilon }_{0}\alpha \alpha^{\prime }}}=\sqrt[{3}]{\frac{q_{N}^{\left(n\right)}q_{B}^{\left(n\right)}}{8{\pi \varepsilon }_{0}\beta \beta^{\prime }}}$ and the distance between the two borons is $|{\vec{\lambda }}_{BB}|=\sqrt[{3}]{\frac{q_{B_{1}}^{\left(m\right)}q_{B_{2}}^{\left(m\right)}}{8{\pi \varepsilon }_{0}\gamma \gamma^{\prime }}}$.

In the B_2_N^(−, 0, +)^ radical, cation and anion forms, the charges of atoms always localize in a definite position in space. In fact, for a charged quantum system, it has been described by the wave function. Thus, the charges distribution with a certain amount in space and different distributions between borons and nitrogen atoms are important for the understanding of real or artifactual SBs problems of radical and ion BNB forms.

## 3. Computational Details

Even though various methods and basis sets (both large and medium) have been employed in this study, among them, the EPR-III and EPR-II basis sets of Barone [40] show accurate results for electrostatic potential (ESP) fitting. EPR-II is a double-ζ basis set with a single set of polarization functions and an enhanced “s” part: (6, 1)/ [41] for H and (10, 5, 1)/ [41,42] for B to F. EPR-III is a triple-ζ basis set including diffuse functions, double d-polarizations, and a single set of f-polarization functions. Also in this case, the s-part is improved to better describe the nuclear region, (6, 2)/[37,38] for H and [40,41,42,43,44] for B up to F.

The active space for the CASSCF methods was composed of all valence electrons and orbitals of these atoms,* i.e.*, 11 active electrons and 12 active orbitals for B_2_N^(0)^ and 10 and 12 electrons for B_2_N^(+)^and B_2_N^(−)^ respectively. In some part of our discussion, the BNB has been optimized via various levels of theory such as CASSCF (11, 12)/cc-pvqz and CASSCF (11, 12)/AUG-cc-pvqz (for radical) and CASSCF (10, 12)/cc-pvqz for cation. Approximation spin orbit coupling between two spin states has been computed during CASSCF calculations [45,46].

A Quadratic CI calculation including single and double substitutions [47] has been used to evaluate various one-electron properties including NBO, bonding analysis, atoms in molecules (AIM) [48], multi-pole moment, natural population analysis, electrostatic potentials, and electrostatic potential-derived charge using the Merz-Kollman-Singh [49], chelp [50], or ChelpG [51].

Polarizabilities and hyper-polarizabilities have been computed by CISD and QCISD and CASSCF methods and double numerical differentiation of energies have been used by with the pol=En only keyword in some cases. The AIM properties have been used to request molecular properties predicted via the theory of atoms in molecules [48]. The AIM keyword is used to compute atomic charges of atoms in molecules, covalent bonds, localized orbitals, and critical points.

Atomic charges have been calculated from electrostatic potentials using a grid-based method, ChelpG, which was developed by Breneman and Wiberg [51]. Atomic charges are fitted to reproduce the molecular electrostatic potential (MESP) at a number of points around the molecule [52,53]. The charge calculation methods based on molecular electrostatic potential (MESP) fitting (including CHELPG) are not well-suited for treating larger systems where some of the innermost atoms are located far away from the points at which the MESP is computed. In such a circumstance, variations of the innermost atomic charges will not lead to significant changes of the MESP outside of the molecule, meaning that the accurate values for the innermost atomic charges are not well-determined by MESP outside of the molecule [52,53].

The representative atomic charges for flexible molecules hence should be computed as average values over several molecular conformations. A number of alternative MESP charge schemes have been developed, such as those employing Connolly surfaces or geodesic point selection algorithms, in order to improve the rotational invariance by increasing the point selection density and reducing anisotropies in the sampled points on the MESP surface. A detailed overview of the effects of the basis set and the Hamiltonian on the charge distribution can be found in reference [54]. CHELPG charges can be computed using the popular *ab initio* quantum chemical packages such as Gaussian or GAMESS-US.

Indeed in our study, because of the large number of calculations in various ESP simulation situations, using expensive basis sets and methods such as MRCI was difficult and also not necessary for CHELPG and ESP calculations. Therefore, with medium methods in terms of computational cost, we have obtained accurate results for our approach. All the calculations were performed using the Gaussian program package [55] and the optimization was done along with a frequency calculation to verify that the geometry was a real minimum without any imaginary frequency.

## 4. Results and Discussion

The radical form of BNB is linear in its ground electronic state (${\tilde{X\;}}^{2}\Sigma_{u}^{+})$) with an orbital occupancy of $1\sigma_{g}^{2},1\sigma_{u}^{2},2\sigma_{g}^{2},3\sigma_{g}^{2},2\sigma_{u}^{2},1\pi_{u}^{4},4\sigma_{g}^{2},3\sigma_{u}^{1}$ while the lowest electronically excited state is predicted to be ${\tilde{A}}^{2}\Sigma_{\left(g\right)}^{+}$, with an orbital occupancy of $1\sigma_{g}^{2},1\sigma_{u}^{2},2\sigma_{g}^{2},3\sigma_{g}^{2},2\sigma_{u}^{2},1\pi_{u}^{4},4\sigma_{g}^{1},3\sigma_{u}^{2}$.

Although the geometric data in Table 1 show boron-nitrogen bonds within a molecule that differ from each other by less than 0.001 angstrom, the BNB in the three radical, cation and anion forms in the ground state are linear. Moreover, the other structures have angles that differ from 180 degrees by less than a degree or in many cases less than a tenth of a degree and the reason depend on the methods used.

In addition, the ${\tilde{\;B}}^{2}\prod_{g}$ exited state with an orbital occupancy of $1\sigma_{g}^{2},1\sigma_{u}^{2},2\sigma_{g}^{2},3\sigma_{g}^{2},2\sigma_{u}^{2},\pi_{u}^{4},4\sigma_{g}^{2},1\pi_{g}^{1}$ (above the ${\tilde{A\;}}^{2}$) is subject to the Renner-Teller effect and further exited states depend on the ${(}^{4}\prod_{g})\;$ of triplet form. The geometric structures and electronic energies in ground and exited states are listed in Table 1.

In addition, NBO, atomic occupancies, Fock Matrix and IFCC [*F* (Δ), *F* (θ)] are listed in Table 2 and Table 3. The total energies of |α〉 and |β〉 spin orbitals for the ground state of strata/stratum, formed with symmetry *C*_2*V*_/*C*_∞*V*_ (global minima of ${\tilde{X\;}}^{2}$Σ^+^), are E(|α〉)=−34.87083 and E(|β〉)=−34.15046 Hartree respectively, while these energies for *C*_∞*V*_/*D*_∞*h*_ (local minima) are E(|α〉)=−34.87079 and E(|β〉)=−34.15042, respectively. Although the energy of ${\tilde{A}}^{2}\Sigma_{\left(g\right)}^{+}$ state is near the ground state, this excited configuration does not contribute to the ground state wave function.

**Figure 1:**
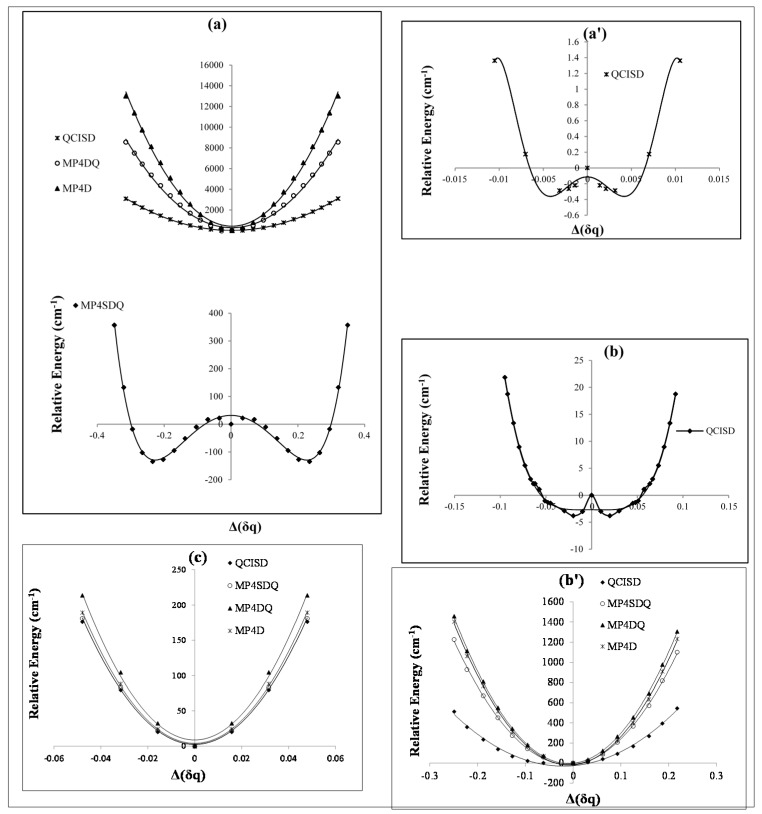
Relative energies of B_2_N^(−, 0, +)^ *versus* B-N-B bond distance in various level of methods (**a, a'**) cation; (**b, b'**) radical and (**c**) anion.

In the symmetric $D_{\infty h}$ geometry, the unpaired electron of BNB is delocalized, while in the asymmetric $C_{\infty v}$ geometry it is localized on either one of the B atoms. Broken symmetry $C_{\infty v}$ structures will be stabilized by this interaction relative to the symmetric $D_{\infty h}$ geometry. Physically, the second-order Jahn-Teller interaction permits the unpaired electron to localize on a single boron atom, rather than being delocalized. The other two, which correspond to localizing the unpaired electron on one or both of the boron atoms (when the bond lengths are unequal), do not transform as an irreducible representation of $D_{\infty h}$ for BNB radical via any changing of charges on N and either one of the B atoms (Figure 1a,a',b).

When the molecule has $D_{\infty h}$ symmetry, the real wave-function must transform as an irreducible representation of the $D_{\infty h}$ point group. However, when the two B–N bonds are asymmetrically stretched, $“4\sigma_{g}^{2}”$ and $“3\sigma_{u}^{1}”$ become near degenerate, while 6σ and 7σ MOs have the same symmetry. Because of this vicinity, the singly excited state of [core]6σ,7σ has a rather strong interaction with single and triple excitations. It is known however, that approximate electronic structure methods could suffer from an artifactual symmetry-breaking effect which would thus be confused as a real Jahn-Teller distortion. DFT methods such as B3LYP incorrectly underestimate the second-order Jahn-Teller distortion which leads the B3LYP calculations to predict a symmetric structure with too much high frequency for the anti-symmetric stretch. However, at this point, the “UHF” solution for the ground state wave-function exists [23].

**Table 1:** Geometric and electronic structures of B_2_N^(−,0,+)^ in ground and exited states.

State	$E_{e}(Hartree)$	|α〉Configuration	|β〉Configuration	$r_{e}\left(B_{1}N\right)$	$A_{1}(2,1,3,-2,-1)$
(*N_e_)		Total Energy of |α*〉	Total Energy of |β〉*, Virtual **	$r_{e}\left(NB_{2}\right)$	$A_{2}(2,1,3,-1,-2)$
		(Homo−Lumo) **			
$\tilde{X\;}$ ^2^Σ^+^	$-104.0781959{\;}^{a}$	$\left[A\right],\pi^{4},\sigma^{2},\sigma^{1}|\alpha \rangle =-0.44641^{\;a}$	$\left[B\right]\sigma^{1}|\beta \rangle =-0.26180{\;}^{a}$	$1.3189^{\;a}$	$A_{1}=179.9499{\;}^{a}$
$\left(C_{\infty v}\right)$	$-104.0820328^{\;a^{\prime }}$	$*E\left(|\alpha \rangle \right)(-34.87083){\;}^{a}$	$*{\left(-34.15046\right)}^{\;a}$	$1.3185{\;}^{a}$	$A_{2}=179.9602{\;}^{a}$
(*17e)	$-104.0754917{\;}^{a^{\prime \prime }}$	$**{\left(-0.48614\right)}^{\;a}$	$**(-0.17697){\;}^{a}$		
${\tilde{X\;}}^{2}\Sigma_{u}^{+}$	$-104.0781959{\;}^{b}$	$\left[A^{\prime }\right]\pi_{u}^{4},4\sigma_{g}^{2},3\sigma_{u}^{1}|\alpha \rangle =-0.44641{\;}^{b}$	$\left[B^{\prime }\right],4\sigma_{g}^{1}|\beta \rangle =-0.26180{\;}^{b}$	$1.3187{\;}^{b}$	$A_{1}=180.0{\;}^{b}$
$\left(D_{\infty h}\right)$		$*(-34.87079){\;}^{b}$	$*(-34.15042){\;}^{b}$	$1.3187^{\;b}$	$A_{2}=180.0{\;}^{b}$
		$**(-0.48614){\;}^{b}$	$**(-0.17697){\;}^{b}$		
${\tilde{X\;}}^{2}\Sigma_{u}^{+}$	$-103.6396773^{\;d}$	$\left[A^{\prime }\right]\pi_{u}^{4},4\sigma_{g}^{2},3\sigma_{u}^{1}|\alpha \rangle =-\;0.42477{\;}^{d}$	$\left[B^{\prime }\right],4\sigma_{g}^{1}|\beta \rangle$=-0.24844 *^d^*	1.3176 *^d^*	$A_{1}=180.0^{\;d}$
$\left(D_{\infty h}\right)$		$*(-34.74888){\;}^{d}$	$*(-34.06924){\;}^{d}$	$1.3176{\;}^{d}$	$A_{2}=180.0{\;}^{d}$
(*17e)		$**(-0.49245){\;}^{d}$	** $\;{\left(-0.18742\right)}^{d}$		
${\tilde{X\;}}^{2}\Sigma_{u}^{+}$	$-104.159145{\;}^{f}$	$\left[A^{\prime }\right]\pi_{u}^{4},4\sigma_{g}^{2},3\sigma_{u}^{1}|\alpha \rangle =\;-0.44701^{\;f}$	$\left[B^{\prime }\right],4\sigma_{g}^{1}|\beta \rangle =-0.26341$	$1.3275{\;}^{f}$	$A_{1}=180.0{\;}^{f}$
$\left(D_{\infty h}\right)$	$-104.1355512^{\;n}$	*(−34.86501)	*(−34.14329)	1.3275	$A_{2}=180.0$
		**(−0.48249)	**(−0.17779)		
${\tilde{A\;}}^{2}\Sigma_{g}^{+}$	$-104.1047582{\;}^{k}$	$\left[A^{\prime }\right]\pi_{u}^{4},4\sigma_{g}^{1},3\sigma_{u}^{2}|\alpha \rangle =-0.44638^{\;u}$	$\left[A^{\prime }\right]\pi_{u}^{4},\;4\sigma_{u}^{1},=-0.26134$	$1.3154{\;}^{k}$	$A_{1}=180.0{\;}^{k}$
(*17e)	$-104.0781729^{\;K^{\prime }}$	$*(-34.8744){\;}^{u}$	*(−34.15408)	$1.3154{\;}^{k}$	$A_{2}=180.0{\;}^{k}$
		$**(-0.48626){\;}^{u}$	**(−0.17666)		
${\tilde{\;B}}^{4}\prod_{g}$	$-104.0297022^{\;h}$	$\left[A^{\prime }\right]\pi_{u}^{4},\pi_{g,}^{2}\sigma_{g}^{1}|\alpha \rangle =-0.26899{\;}^{h}$	$\left[A^{\prime }\right],\pi_{u}^{2}|\beta \rangle -0.49646$	$1.3079{\;}^{h}$	$A_{1}=180.0{\;}^{h}$
(*17e)	$-104.0141196{\;}^{a}$	$*(-35.04484){\;}^{h}$	*(−33.74801)	$1.3079^{\;h}$	$A_{2}=180.0{\;}^{h}$
		$*(-0.30125){\;}^{h}$	**(−0.50595)		
${\tilde{X\;}}^{1}\Sigma_{g}^{+}$	$-104.1965676{\;}^{a}$	$\left[A^{\prime }\right]1\pi_{u}^{4},4\sigma_{g}^{2},3\sigma_{u}^{2}=-0.13904{\;}^{a}$		$1.3291{\;}^{a}$	$A_{1}=179.9322^{\;a}$
(*18e)	$-104.2019114{\;}^{a^{\prime }}$	$*E\left(|\alpha \rangle \right)=-32.48647{\;}^{a}$		1.3291	$A_{2}=179.9462$
	$-104.1950169^{\;a^{\prime \prime }}$	**−0.36286			
${\tilde{X\;}}^{1}\Sigma_{g}^{+}$	$-104.1965676{\;}^{b}$	$*E\left(|\alpha \rangle \right)=-32.48644{\;}^{b}$		$1.3291{\;}^{b}$	$A_{1}=180.0{\;}^{b}$
(*18e)				1.3291	$A_{2}=180.0$
${\tilde{X\;}}^{1}\Sigma_{g}^{+}$	$-104.1145486{\;}^{c}$	$\left[A^{\prime }\right]\pi_{u}^{4},\sigma_{g}^{2},\sigma_{u}^{2}=-0.13454^{\;c}$		$1.3459{\;}^{c}$	$A_{1}=179.8967{\;}^{c}$
(*18e)	$-104.1162881^{\;c^{\prime }}$			$1.3459{\;}^{c}$	$A_{2}=179.9181{\;}^{c}$
	$-104.112568{\;}^{c^{\prime \prime }}$				
${\tilde{\;A}}^{3}\Pi_{u}$	$-104.0884685{\;}^{a}$	**[C]**$\pi_{u}^{4},3\sigma_{u}^{1},1\pi_{g}^{1}|\alpha \rangle$=$-0.03986{\;}^{h}$	$[C^{\prime }]\pi_{u,}^{4},4\sigma_{g}^{1}|\beta \rangle$ = $-0.01559{\;}^{h})$	$1.3422^{\;h}$	$A_{1}=180.0{\;}^{h}$
(*18e)	$-104.1164696{\;}^{h}$	$*E\left(|\alpha \rangle \right)=-32.79721^{\;h}$	$*E\left(|\beta \rangle \right)=-32.26335{\;}^{h}$	1.3422	$A_{2}=180.0$
	$-104.0809447{\;}^{a^{\prime }}$	$**-0.25941^{\;h}$	$**-0.14777^{\;h}$		
	$-104.0792592^{\;a^{\prime \prime }}$				
${\tilde{X\;}}^{1}\Sigma_{g}^{+}$	$-103.7454365{\;}^{a}$	$[D](1\pi_{u}^{4}),(4\sigma_{g}^{2}$=$-0.57788{\;}^{a})$		$1.2938{\;}^{a}$	$A_{1}=180.7911^{\;a}$
(*16e)	$-103.8054934{\;}^{a^{\prime }}$	$*E\left(|\alpha \rangle \right)=-36.52419{\;}^{a}$		$1.2938{\;}^{a}$	$A_{2}=180.6276{\;}^{a}$
	$-103.7545006{\;}^{a^{\prime \prime }}$	$**-0.17926^{a}$			
${\tilde{X\;}}^{1}\Sigma_{g}^{+}$	$-103.6023122{\;}^{m}$	$[D](1\pi_{u}^{4}),(4\sigma_{g}^{2}$=$-0.57803{\;}^{g})$		$1.3156^{\;m\;}$	$A_{1}=179.981^{m}$
(*16e)	$-103.3012055{\;}^{g}$	$*E\left(|\alpha \rangle \right)=-36.51985{\;}^{g}$		$1.3156{\;}^{m}$	$A_{2}=179.985^{m}$
	$-103.8377401{\;}^{f}$	$**-0.17944{\;}^{g}$		$1.3003{\;}^{h}$	
	$-103.7903312^{\;h}$			$1.3004^{\;h}$	
$\tilde{B\;}$ ^3^Σ_g_	$-103.7609202{\;}^{a}$	$\{\left[A^{\prime }\right]4\sigma_{g}^{1},3\sigma_{u}^{1},\pi_{u}^{4},\}{\;}^{a}$	$\{\left[A^{\prime }\right]\;,\pi_{u}^{2}|\beta \rangle =-0.75222\}{\;}^{a}$	$1.2976{\;}^{a}$	$A_{1}=180.0^{\;a}$
(*16e)	$-103.7767141{\;}^{h}$	$\pi_{u}^{2}|\alpha \rangle =-0.74323{\;}^{a}$	$*E\left(|\beta \rangle \right){\left(-35.72475\right)}^{a\;}$	$1.2976^{\;a}$	$A_{2}=180.0$
	$-103.7628505{\;}^{a^{\prime }}$	$*E\left(|\alpha \rangle \right)(-37.33372){\;}^{a}$	$**(-0.52131){\;}^{a}$		
	$-103.7590557{\;}^{a^{\prime \prime }}$	$**(-0.53385){\;}^{a}$			

^(a)^ QCISD/EPR-II, ^(a'')^ MP_4_D/EPR-III//QCISD/EPR-III, ^(m)^ CASSCF ^(a'')^ MP_4_SDQ/EPR-III//QCISD/EPR-III, ^(d)^ CASSCF (11, 12)/UHF, ^(g)^ CASSCF(10,12)rohf AUG-cc-pvqz, ^(b)^ QCISD/EPR-III($\theta_{const}=180.0)$, $\left[\;A\;\right]:1\sigma^{2},2\sigma^{2},3\sigma^{2},4\sigma^{2},5\sigma^{2}$, ^(c)^ QCISD/EPR-II, $\left[A^{\prime }\right]:1\sigma_{g}^{2},1\sigma_{u}^{2},2\sigma_{g}^{2},3\sigma_{g}^{2},2\sigma_{u}^{2}$; ^(c')^ MP_4_D/EPR-II//QCISD/EPR-II, $\left[B\;\right]:\;\sigma^{1},\sigma^{1},\sigma^{1},\sigma^{1},\sigma^{1},\pi^{2}$; ^(c'')^ MP_4_SDQ/EPR-II//QCISD/EPR-II, [$B^{\prime }$]: $1\sigma_{g}^{1},2\sigma_{g}^{1},1\sigma_{u}^{1},3\sigma_{g}^{1},2\sigma_{u}^{1},1\pi_{u}^{2}$, ^(f)^ CBS-lq, ^(h)^ QCISD(T)/EPR-III, [C]: $1\sigma_{g}^{2},1\sigma_{u}^{2},2\sigma_{g}^{2},3\sigma_{g}^{2},2\sigma_{u}^{2},4\sigma_{g}^{2}$, $\left[C^{\prime }\right]:1\sigma_{g},1\sigma_{u},2\sigma_{g},3\sigma_{g},2\sigma_{u}$, ^(n)^ CBS4O, ^(u)^ TD/EPR-II ^(k)^ TD/EPR-III//QCISD (T)/EPR-III, ^(*k'*)^ TD/EPR-III//QCISD /EPR-III, [D]: $1\sigma_{g}^{2},2\sigma_{g,}^{2}1\sigma_{u}^{2},3\sigma_{g}^{2},2\sigma_{u}^{2}$. Total energy of |β〉 and the energy of virtual orbital are shown with “*” and “**” symbols, respectively.

**Table 2:** NBO, electric potential, gradient of electric potential (ℒ), atomic occupancies, and Fock Matrix data of B_2_N^(−, 0**, +)**^ in ground and exited states.

State	$B^{Ep_{1}}–N^{Ep_{2}}–B^{Ep_{3}}$	$ℒ=\frac{E_{P{(}_{B})}-E_{P{(}_{N})}}{R(BN)}$	Hybrids Coefficient&	$E_{acceptor_{\left(j\right)}}-E_{Donor_{\left(i\right)}}$	Atomic Occupancies
(*N_e_)				$*Fock\;Matrix\;(F_{i,j},a.u.)$	
${\tilde{X\;}}^{2}\Sigma_{u}^{+}$	$N^{Ep_{2}}=-18.3638{\;}^{a}$	(5.32), (5.32)	${|\psi \rangle }_{BD\left(1\right)}=0.91SP_{N_{1}}^{1.01}+0.41\;SP_{B_{2}}^{2.46^{a}}$	${|\psi \rangle }_{BD^{*}\left(2\right)}-{|\psi \rangle }_{BD\left(1\right)}=2.01$	$|\alpha \rangle N:2s^{0.77}2P_{x}^{0.84}2P_{y}^{0.84}2P_{z}^{0.87}$
(*17e)	$B^{Ep_{1}}=B^{Ep_{3}}-11.3464^{\;a}$		${|\psi \rangle }_{BD(2)}=0.96SP_{N_{1}}^{1.0}+0.29SP_{B_{2}}^{1.0^{a}}$	* 0.094	$|\alpha \rangle B:2s^{0.76}2P_{x}^{0.79}2P_{y}^{0.79}2P_{z}^{0.40}$
			${|\psi \rangle }_{BD(3)}=0.96SP_{N_{1}}^{1.0}+0.29SP_{B_{2}}^{1.0^{a}}$	${|\psi \rangle }_{BD^{*}\left(3\right)}-{|\psi \rangle }_{BD\left(1\right)}=1.00$	$|\beta \rangle N:2s^{0.80}2P_{x}^{0.86}2P_{y}^{0.86}2P_{z}^{0.83}$
			${|\psi \rangle }_{BD^{*}\left(1\right)}=0.28SP_{N_{1}}^{1.0}-0.95\;SP_{B_{2}}^{1.0^{a}}$	* 0.089	$|\beta \rangle B:2s^{0.50}2P_{x}^{0.69}2P_{y}^{0.69}2P_{z}^{0.16}$
			${|\psi \rangle }_{BD^{*}\left(2\right)}=0.42SP_{N_{1}}^{1.01}-0.90\;SP_{B_{3}}^{0.95^{a}}$	${|\psi \rangle }_{BD^{*}\left(1\right)}-{|\psi \rangle }_{BD\left(2\right)}=0.7$	
			${|\psi \rangle }_{BD^{*}\left(3\right)}=0.70SP_{B_{2}}^{0.97}-0.70\;SP_{B_{3}}^{0.97^{a}}$	* 0.029	
${\tilde{X\;}}^{1}\Sigma_{g}^{+}$	$N^{Ep_{2}}=-18.6013^{\;a}$	(5.25), (5.25)	${|\psi \rangle }_{BD\left(1\right)}=0.90SP_{N_{1}}^{1.01}+0.43\;SP_{B_{2}}^{2.26^{a}}$	${|\psi \rangle }_{BD^{*}\left(3\right)}-{|\psi \rangle }_{BD\left(1\right)}=1.94$	$|\alpha \rangle ,\beta \rangle N:2s^{1.59}2P_{x}^{1.70}2P_{y}^{1.70}2P_{z}^{1.66}$
(*18e)	$B^{Ep_{1}}=B^{Ep_{3}}-11.6190^{\;a}$		${|\psi \rangle }_{BD(2)}=0.66SP_{N_{1}}^{1.0}+0.75SP_{B_{2}}^{1.0^{a}}$	* 0.107	$|\alpha \rangle ,\beta \rangle B:2s^{0.97}2P_{x}^{0.14}2P_{y}^{0.14}2P_{z}^{0.37}$
			${|\psi \rangle }_{BD(3)}=0.95SP_{N_{1}}^{1.0}+0.32SP_{B_{2}}^{1.0^{a}}$	${|\psi \rangle }_{BD^{*}\left(1\right)}-{|\psi \rangle }_{BD\left(1\right)}=0.73$	
			${|\psi \rangle }_{BD^{*}\left(1\right)}=0.44SP_{N_{1}}^{1.01}-0.90\;SP_{B_{2}}^{2.24^{a}}$	*0.021	
			${|\psi \rangle }_{BD^{*}\left(2\right)}=0.75SP_{N_{1}}^{1.0}-0.67\;SP_{B_{3}}^{1.0^{a}}$	${|\psi \rangle }_{BD^{*}\left(3\right)}-{|\psi \rangle }_{BD\left(3\right)}=1.94$	
			${|\psi \rangle }_{BD^{*}\left(3\right)}=0.32SP_{N_{1}}^{1.0}-0.94\;SP_{B_{3}}^{1.0^{a}}$	* 0.107	
$\tilde{B\;}$ ^3^Σ^+^	$N^{Ep_{2}}=-18.10657{\;}^{h}$	(5.43), (5.43)	${|\psi \rangle }_{BD\left(1\right)}=0.90SP_{N_{1}}^{1.0}+0.42\;SP_{B}^{0.72^{\;h}}$	${|\psi \rangle }_{BD^{*}\left(3\right)}-{|\psi \rangle }_{BD\left(1\right)}=1.98$	$|\alpha \rangle N:2s^{0.78}2P_{x}^{0.81}2P_{y}^{0.81}2P_{z}^{0.87}$
(*16e)	$B^{Ep_{1}}=B^{Ep_{3}}-11.0485^{\;h}$		${|\psi \rangle }_{BD(2)}=0.96SP_{N_{1}}^{1.0}+0.26SP_{B}^{1.0^{h}}$	* 0.119	$|\alpha \rangle B:2s^{0.72}2P_{x}^{0.92}2P_{y}^{0.92}2P_{z}^{0.44}$
			${|\psi \rangle }_{BD(3)}=0.96SP_{N_{1}}^{1.0}+0.26SP_{B}^{1.0^{h}}$	${|\psi \rangle }_{BD^{*}\left(1\right)}-{|\psi \rangle }_{BD\left(2\right)}=0.77$	$|\beta \rangle N:2s^{0.77}2P_{x}^{0.87}2P_{y}^{0.87}2P_{z}^{0.86}$
			${|\psi \rangle }_{BD^{*}\left(1\right)}=0.42SP_{N_{1}}^{1.0}-0.90\;SP_{B_{2}}^{1.0^{h}}$	* 0.028	$|\beta \rangle B:2s^{0.10}2P_{x}^{0.62}2P_{y}^{0.62}2P_{z}^{0.08}$
			${|\psi \rangle }_{BD^{*}\left(2\right)}=0.26SP_{N_{1}}^{1.01}-0.96\;SP_{B_{3}}^{0.95^{h}}$	${|\psi \rangle }_{BD^{*}\left(1\right)}-{|\psi \rangle }_{BD\left(2\right)}=0.7$	
			${|\psi \rangle }_{BD^{*}\left(3\right)}=0.42SP_{N_{1}}^{1.0}-0.90\;SP_{B_{3}}^{0.72^{h}}$	* 0.029	
${\tilde{\;A}}^{3}\Pi_{u}$	$N^{Ep_{2}}=-18.5665^{\;h}$	(5.19), (5.19)	${|\psi \rangle }_{BD\left(1\right)}=0.91SP_{N_{1}}^{1.0}+0.40\;SP_{B_{2}}^{1.62^{h}}$	${|\psi \rangle }_{BD^{*}\left(3\right)}-{|\psi \rangle }_{BD\left(1\right)}=1.94$	$|\alpha \rangle N:2s^{0.75}2P_{x}^{0.81}2P_{y}^{0.60}2P_{z}^{0.86}$
(*18e)	$B^{Ep_{1}}=B^{Ep_{3}}-11.5955^{\;h}$		${|\psi \rangle }_{BD(2)}=0.97SP_{N_{1}}^{1.0}+0.26SP_{B_{2}}^{1.0^{h}}$	* 0.107	$|\alpha \rangle B:2s^{0.74}2P_{x}^{0.09}2P_{y}^{0.70}2P_{z}^{0.43}$
			${|\psi \rangle }_{BD(3)}=0.91SP_{N_{1}}^{1.0}+0.40SP_{B_{3}}^{1.62^{h}}$	${|\psi \rangle }_{BD^{*}\left(4\right)}-{|\psi \rangle }_{BD\left(1\right)}=1.01$	$|\beta \rangle N:2s^{0.80}2P_{x}^{0.87}2P_{y}^{0.90}2P_{z}^{0.86}$
			${|\psi \rangle }_{BD(4)}=0.70SP_{B_{2}}^{0.51}+0.70SP_{B_{3}}^{0.51}$	* 0.064	$|\beta \rangle B:2s^{0.46}2P_{x}^{0.06}2P_{y}^{0.04}2P_{z}^{0.16}$
			${|\psi \rangle }_{BD^{*}\left(1\right)}=0.4SP_{N_{1}}^{1.0}-0.91\;SP_{B_{2}}^{1.62^{h}}$	${|\psi \rangle }_{BD^{*}\left(1\right)}-{|\psi \rangle }_{BD\left(2\right)}=0.73$	
			${|\psi \rangle }_{BD^{*}\left(2\right)}=0.26SP_{N_{1}}^{1.0}-0.97\;SP_{B_{3}}^{1.0^{h}}$	* 0.021	
			${|\psi \rangle }_{BD^{*}\left(3\right)}=0.40SP_{N_{1}}^{1.0}-0.91\;SP_{B_{3}}^{1.62^{h}}$		
			${|\psi \rangle }_{BD^{*}\left(4\right)}=0.70SP_{B_{2}}^{0.51}-0.70\;SP_{B_{3}}^{051^{h}}$		

The Fock Matrix $\left(F_{i,j},a.u.\right)$ data have been shown with star symbol “*”. ^(a)^ QCISD/EPR-III, ^(h)^ QCISD(T)/EPR-III.

**Table 3:** Isotropic Fermi contact coupling (IFCC) of B_2_$N^{\left(0\right)}$ in ground (${\tilde{X\;}}^{2}\Sigma_{u}^{+}$) and exited (${\tilde{\;B}}^{4}\prod_{g}$) states.

State	$\Delta =r_{2}-r_{1}$	IFCC[f(Δ)]	$\Delta \theta =\theta_{1}-\theta_{1}$	IFCC[f(θ)]
(*N_e_)	$\theta_{cons}=180.0$	$N,B_{1}$**,**$\;B_{2}$	$r_{cons}=1.3176$	$N,B_{1}$**,**$\;B_{2}$
${\tilde{X\;}}^{2}\Sigma_{u}^{+}$ (*17e)	Δ =0.000	−29.81, 428.6, 428.6	Δθ=0.0	−29.81, 428.6, 428.6
	Δ =0.010	−29.76, 386.2, 469.7	Δθ=2.0	−29.81, 428.71, 428.71
	Δ =0.020	−29.11, 348.2, 508.1	Δθ=3.0	−29.81, 428.77, 428.77
	Δ =0.030	−28.02, 316.1, 541.9	Δθ=5.0	−29.81, 428.95, 428.95
	Δ =0.040	−26.64, 290.3, 570.2	Δθ=10.0	−29.83, 429.84, 429.84
	Δ =0.050	−25.12, 270.7, 592.6	Δθ=20.0	−29.87, 433.44, 433.44
	Δ =0.060	−23.54, 256.7, 609.5	Δθ=30.0	−29.86, 439.68, 439.68
	Δ =0.070	−21.98, 247.6, 621.3	Δθ=40.0	−29.61, 448.75, 448.75
	Δ =0.080	−20.46, 242.7, 628.7	Δθ=50.0	−28.85, 460.82, 460.82
	Δ =0.082	−20.10, 242.1, 629.8	Δθ=60.0	−27.16, 475.86, 475.86
	Δ =0.085	−19.72, 241.6, 630.9	Δθ=70.0	−24.06, 493.31, 493.31
	Δ =0.086	4.35, 60.3, 899.9	Δθ=80.0	−18.57, 512.55, 512.55
	Δ =0.087	4.46, 59.4, 901.2	Δθ=90.0	−10.65, 533.75, 533.75
	Δ =0.090	4.79, 56.6, 904.7		
${\tilde{\;B}}^{4}\prod_{g}$	Δ =0.000	20.26, 377.8, 377.8		
	Δ =0.010	20.26, 381.2, 377.9		
	Δ =0.020	20.26, 384.5, 377.9		
	Δ =0.030	20.28, 387.8, 377.9		
	Δ =0.040	20.30, 391.2, 377.9		
	Δ =0.050	20.30, 394.4, 377.9		
	Δ =0.060	20.38, 397.8, 377.9		
	Δ =0.070	20.44, 401.0, 377.8		
	Δ =0.080	20.50, 404.27, 377.7		

It is prudent to employ a highly correlated method which can use a large number of reference determinants to recover dynamic and static correlations. In this work, the results of EPR-III basis sets are monotonous through the comparison between different situations. Although (in Table 1) the difference between the two positions of global minima and local minima for both |α〉 and |β〉 is 8.77 cm^−1^, our calculations show that the total energies for both of them are the same (*i.e.*, −104.0781959). This is due to the fact that the spin orbital energies are related to the small bending angles of A_1_ and A_2_ which have an extremely low bending frequency (70 cm^−1^). Harmonic frequencies were determined at the QCISD/EPR-III//prop=EPR and characterized by 228.79 cm^−1^ ($\vartheta_{1}=\vartheta_{1}^{\prime },\;$ bending mode $“\pi_{u}”$), 1178.64 cm^−1^ ($\vartheta_{2}$, symmetric stretching “$\sigma_{g}$”) and 2146.42 ($\vartheta_{3}$, asymmetric stretching “$\sigma_{u}$”). The IR and Raman intensities for $\vartheta_{3}$ are 10165.0 and 0.00, respectively, while the $“\vartheta_{2}”$ mode has intensity in Raman (51.0) but zero intensity in the IR region.

As it is shown in Table 1, the energy difference between two states of (${\tilde{A\;}}^{2}\Sigma_{g}^{+}$) and (${\tilde{X\;}}^{2}\Sigma_{u}^{+})$ {(k − a) and (k – ${\text{ k}}^{\prime }$)} are 5829.75 cm^−1^ and 5834.79 cm^−1^, respectively. Those values are close to the photoelectron spectroscopy calculation results which *Asmis et al.* have shown [2]. They have discussed that the signal observed in the 355 nm and 266 nm photoelectron spectra of $\;B_{2\;}N^{-}$ has been indicated as due to a photodetachment from the ${\tilde{X}}^{1}\;\Sigma_{g}^{+}\;)$ to the ground and lowest excited state of neutral B_2_N {${\tilde{X}}^{2}\;\Sigma_{u}^{+}\;)$ and $\left(\;{\tilde{A}}^{2}\;\Sigma_{g}^{+}\;\right)$ with a linear symmetry and assigned to the ${\tilde{X}}^{1}\;\Sigma_{g}^{+}\;\to {\tilde{X}}^{2}\;\Sigma_{u}^{+}+\;e^{-}$ and ${\tilde{X}}^{1}\;\Sigma_{g}^{+}\;\to {\tilde{A}}^{\;2}\;\Sigma_{g}^{+}+\;e^{-}$ transitions {the $\left(\;{\tilde{A}}^{2}\;\Sigma_{g}^{+}\;\right)$ term energy *T*_0_ is 0.785 eV or 6331.77 cm^−1^}.

The $“1\pi_{u}”$ orbital is a bonding combination of all $2p{\;}_{\pi }$ orbitals on all three atoms, while $“4\sigma_{g}^{2}”$ and $“3\sigma_{u}^{1}”$ orbitals are close lying and not strongly bonding in character. The small separation of these two orbitals accounts for the small energy required to promote the molecule from the ground state to the first exited state at 5829.75 cm^−1^. The difference between (k − a) and (k − $k^{\prime }$) is ≈ 5 cm^−1^ which is near 8.77 cm^−1^ (different between $C_{2V}/C_{\infty V}$ *and* $C_{\infty V}/D_{\infty h}$ of strata/stratum). In addition ${\tilde{\;B}}^{2}\prod_{g}$ (exited state above the ${\tilde{A\;}}^{2}\Sigma_{g}^{+}$) is subject to the Renner-Teller effect, leading to a complicated pattern of bending vibrational levels. Our calculation shows the existence of a larger gap between $3\sigma_{u}$ and $1\pi_{g}$ orbitals, thereby placing the transition to $\left[A^{\prime }\right]\pi_{u}^{4},4\sigma_{g}^{2},1\pi_{g}^{1}$ with ${\tilde{\;B}}^{2}\prod_{g}$ state much higher in energy. Analysis of the vibronic structure of the ${\tilde{\;B}}^{2}\prod_{g}-{\tilde{(X\;}}^{2}\Sigma_{u}^{+})$ band system shows the transition to ${\tilde{B}}^{2}\prod_{g}$ at 19,452 cm^−1^. Nevertheless, the nonbonding character of $1\pi_{g}$ and $3\sigma_{u}$ orbitals implies no significant change in B–N bond lengths in this transition, as it is observed by Ding *et al.* [56]. Therefore, in spite of ${\tilde{A}}^{2}\Sigma_{\left(g\right)}^{+}$, the “${\tilde{\;B}}^{2}\prod_{g}$” excited configuration does contribute to the ground state wave function as a subject of Renner-Teller effect.

It is notable that the electron configuration sequences for the |α〉 and |β〉 situations are not the same and as shown in Table 1. These sequences correspond to $\left[A^{\prime }\right]$ and $\left[B^{\prime }\right]$ for |α〉 and |β〉, respectively, which result the sequences of $“1\sigma_{g}>1\sigma_{u}>2\sigma_{g}”$ for |α〉 and $“1\sigma_{g}>2\sigma_{g}>1\sigma_{u}”$ for |β〉. They indicate the relation between Centro-symmetric and electron correlations in various levels from UHF and DFT to the CASSCF and MRCI methods for the magnitude of the barrier energies. As a result, if the molecule has a barrier at the Centro-symmetric structure, the barrier is not extraordinarily high, and if a high barrier were present, the vibrational level of the ground state would be thermally populated and transitions from this level would be sharp. Ding shows that in order to decide whether a barrier exists at the Centro-symmetric configuration, the energies of the $\left(\vartheta_{3}\right)$ levels in the ground state would have to be measured, and the resulting set of levels used to deduce the potential function along the anti-symmetric stretching coordinate, *Q_3_*. For even values of *v*_3_, this would be possible in principle by either dispersed fluorescence or stimulated emission pumping spectroscopy. In practice, however, it appears that these levels will be difficult to reach owing to poor Franck-Condon factors. For odd values of $\vartheta_{3}$, a direct infrared absorption study provides the best method for the accurate measurement of those levels [56].

Although the observation of excitations involving uneven quanta of the anti-symmetric stretching mode, ***v*_3_**, indicates a breakdown of the Franck–Condon (FC) approximation, it cannot be the only results from Herzberg–Teller vibronic coupling between the (${\tilde{X}}^{2}\;\Sigma_{u}^{+})$) and ($\left({\tilde{A}}^{2}\;\Sigma_{g}^{+}\right)$) states involving the ***v*_3_** mode.

In Table 2, the NBO calculation shows that the 2*s*_N_ orbital is considered to be primarily core-like, forming the $3\sigma_{g}$ orbital, though, of course, some mixing of the 2*s*_N_ orbital into the other $\sigma_{g}$ orbitals ix expected. NBO analysis of the orbital containing the unpaired electron in BNB shows that most of the spin density is located in the boron sp orbitals. The boron atomic orbitals are best described as a “sp” hybrid, directed away from the nitrogen atom however, bonding with respect the σP orbital on the nitrogen atom. The $2\sigma_{u}$ orbital is a bonding combination of the 2pσ orbital on the central nitrogen atom with 2spσ hybrid orbitals on the two borons. The $1\pi_{u}$ orbital is a bonding combination of all 2pπ orbitals on all three atoms, while $“4\sigma_{g}”$ and $“3\sigma_{u}”$ orbitals are close lying and not strongly bonding in character.

The hyperfine parameters were calculated for the linear geometry with a bond length of 1.3176 Å via a CASSCF optimization at several levels of configuration interaction and exited states (Table 3).

$A_{iso}$ in the ground state for boron atoms varies from 428.6 MHz at Δ =0.0 to 241.6 MHz at Δ =0.085 for B1 and from 428.6 MHz at Δ =0.0 to 630.9 MHz at Δ =0.085 for B2 while the nitrogen $A_{iso}$ varies smoothly from −29.81 MHz (Δ =0.0) to −19.72.0 MHz (Δ =0.085). There is a critical point for $A_{iso}$ (both in B and N) between Δ =0.085 and Δ =0.086 where the data reverses to 4.35, 60.3and 899.9 for N, B1 and B2 respectively (Table 3), indicating the symmetry breaking in point charges or distances in this region. The dipolar hyperfine coupling constants exhibited negligible dependence on bound. The highest occupied orbital containing the unpaired electron is $\;\sigma_{u}$ orbital with most of the electron density on the boron atoms. Symmetry constrains this orbital to have only “σP” orbital contributions from nitrogen with no “s” character so that the isotropic hyperfine parameter from nitrogen is small and arises mostly from spin polarization effects.

The molecular isotropic hyperfine $\left(A_{iso}\right)$ values for BNB can be obtained from the experimental “$A_{\|}$” and “$A_{⊥}$”: $A_{iso}=\frac{\left(2A_{⊥}+A_{\|}\right)}{3}=\frac{8\pi }{3}\;g_{e}g_{N}\beta_{e}\beta_{N}<\left(r\right)>$ (10)

As a result, the experimental data are B1 = B2 = 451 and N = −14.0 [4] which are close to our calculated results. Isotropic hyperfine interaction including “**g**” tensor for BNB shows the large boron isotropic amount. Thus, the properties of the **g** tensor eliminate the possibility of a low exited 2Πg state for the radical. The averaged vibrations of B–N bond lengths, IFCC[f(Δ)] and IFCC[f(θ)] can be shown, even if the ground state is precisely linear, quasi-linear or the geometry at the potential minimum went through a symmetry breaking to form a $C_{\infty v}$ structure.

**Figure 2:**
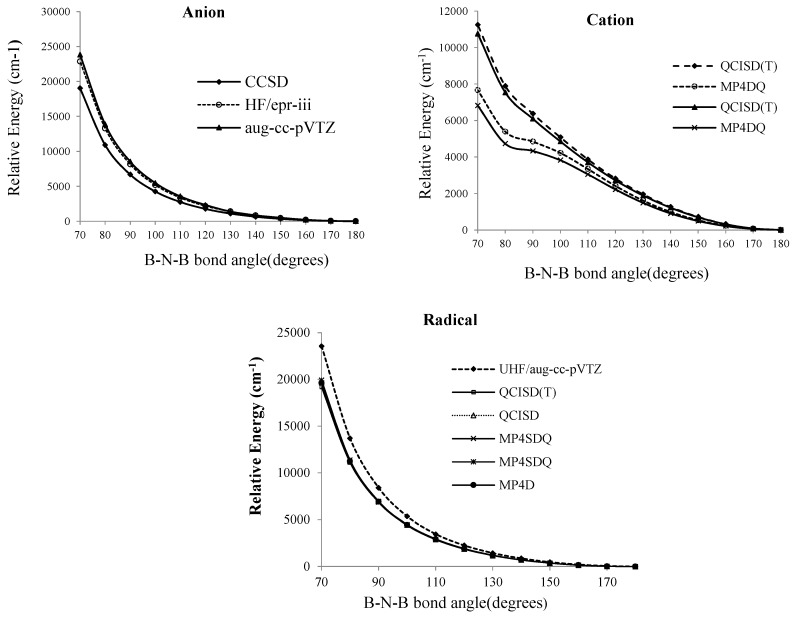
Relative energies of B_2_N^(−, 0, +)^* vs.* B-N-B bond angle in various level of methods for anion, cation and radical forms of BNB respectively

In this study, we have focused on the charge distribution of boron and nitrogen atoms to exhibit the charge breaking (Table 1). As it is shown in Figure 1 and Figure 2, the symmetry breaking in point charge distribution does not only indicate a barrier energy in the regions of 100–160 cm^−1^ [9,22,23] or 20 cm^−1^ [20], but also it creates several SB through the asymmetry stretching (or interaction between asymmetry stretching and bending) with different barrier energies from high to small values (about 5 cm^−1^) (Figure 1).

Therefore, the symmetry-breaking barrier has a dynamic changing with no Centro-symmetric form, and it depends on wave function or charge distribution. Furthermore, a large barrier can be estimated via fixed-node diffusion Monte Carlo methods in which $D_{\infty h}$ and $C_{\infty v}$ ROHF WFs (with symmetric stretching) have energies separated by a gap as big as 0.5 milli Hartree approximately [57]. In the case of $C_{\infty V}$ symmetry solutions, there are two degenerate solutions which correspond to a single electron, localized on either one of the two “different” boron atoms, as can be observed from a natural bond analysis. A linear combination between these two solutions will restore the $D_{\infty h}$ symmetry of the WF, but this WF would be different from the original $D_{\infty h}$ solution where the single electron is delocalized over the two boron atoms [57].

**Figure 3:**
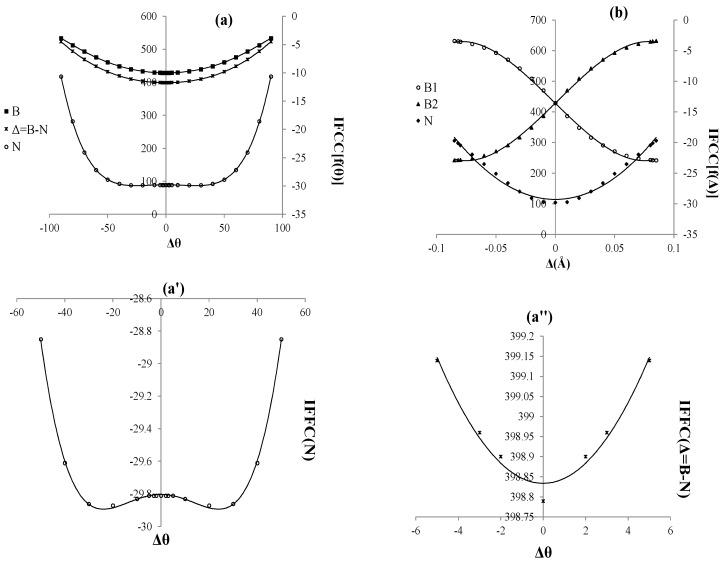
Isotropic Fermi contact coupling (IFCC) of B_2_N^(0)^: (**a**) Function of angles changing; (**b**) Function of distances changing. The axes for nitrogen and borons have different scales; (**a'**) IFCC for Nin a short scale between −30 to −29.8 indicates of IFCC breaking and (**a''**) different IFCC of B and N in a short scale.

As shown in Figure 3, based on Knight’s reports [4] {(in which the variation of energy with bond angle for finding a minimum around (100°) have been discussed}, a cyclic radical or anion B_2_N^(−, 0)^ in our calculations has not been observed, though, for cation, there is a bulge in the curve at 90° in MP_4_DQ and MP_4_DSQ methods which indicates a cyclic B_2_N^+^.

At the SCF level, the lowest energy corresponds to a bent molecule with an angle of 100°, however, for the QCISD (T), MP_4_DQ, MP_4_DSQ and HF/aug-cc-pVTZ calculations (Figure 2) the linear structure clearly has the lowest energies for radical and anion structures. Martin [7] has shown a cyclic B_2_N (^2^B_2_) via reactions of pulsed laser produced boron and nitrogen atoms in a condensed argon stream (at higher laser power reactions) and has discussed that the vibration 882.3 cm^−1^ must be considerably an-harmonic. This possibility receives substantial support from the five combination bands observed in the 3000–6500 cm^−1^ regions*.* The 882.3 cm^−1^ one is assigned to the anti-symmetric B-N stretching fundamental *v_3_*(b_2_) of cyclic B_2_N and the 1998.4 cm^−1^ combination band is the sum of *v*_1_ (a_1_), the symmetric B-N stretching fundamental, and *v*_3_. The difference 1998 − 882 = 1116 cm^−1^ can help to measure the *v*_1_.

The electronic transition energy σ_*g*_ → σ_*u*_ excitation is less than 6000 cm^−1^, which indicates that the higher overtones of the cyclic B_2_N (^2^*B^2^* state) vibrations will display significant vibronic interaction effects. The failure to observe cyclic B_2_N in the ^2^*B^2^* state by ESR [4] is most likely due to the differences in production and relaxation of the energized evaporated species. The fact that the radical BNB might be converted to cation BNB towards the cyclic B2N (due to the laser ionization effect) can be predicted from Martin’s study. In addition, Becker* et al. *[58] used laser ionization mass spectrometry to study the formation of $B_{n}N_{m}^{+}$ clusters ions in laser plasma which resulted in production of BNB cation whereas our calculations resulted in production of cyclic B_2_N^+^.

**Scheme 2:**
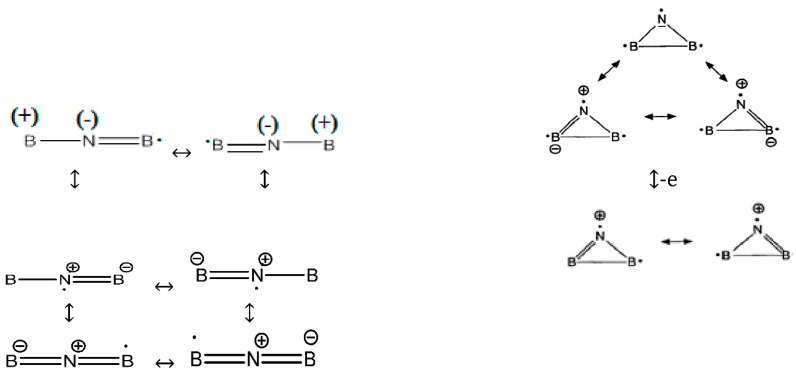
Lewis structures representation of BNB in three forms of radical, anion and cation.

In a simple form, the best Lewis structure representation (Scheme 2) is the pair of resonance structures,* i.e.*, “.B=N=B:” and “: B=N=B.” These structures have a formal +1 charge on nitrogen and a formal −1/2 charge on each boron, but the difference in electronegativity gives rise to a net-negative charge density on the nitrogen atom. Radical of B_2_$N^{\left(0\right)}$ is stabilized by six resonance structures of the linear forms and three resonance structures of cyclic forms (with various distributions) as follows:

Although in some structures of the six resonances, the nitrogen is negative and the two borons are positive, in our ESP calculations for B_2_$N^{\left(0\right)}$ with different levels of theory, nitrogen is always positive and close to zero while the two boron atoms are always negative near zero and the converse appears for the B_2_N^(+)^ species. The atomic charges via 4516 points for one of the ESP fitting at QCISD/EPR-III level of theory for radical shows the values of 0.076055, −0.037580 and −0.038476 for N and two boron atoms, respectively.

All in all, the nuclear hyperfine of various states are shown in Table 3 and Figure 3. The nitrogen and one of the boron atoms in ${\tilde{\;B}}^{4}\prod_{g}$ state are independent of “Δ” during hyperfine coupling calculation. Although these two states ${\tilde{\;B}}^{4}\prod_{g}$ and ground state) can interact along the anti-symmetric stretching as a reason of SB problem in BNB radical, the lack of the “correct” per-mutational symmetry of the wave-functions which arises due to the oversimplification of the wave function is a major reason for spontaneous symmetry breaking.

For the sextuplets contaminating ${\text{C}}_{\infty \text{V}}$ symmetry ^2^Σ^+^: $\;1\sigma^{2},2\sigma^{2},3\sigma^{2},4\sigma^{2},5\sigma^{2},6\sigma^{2},7\sigma^{1},1\pi^{2},1\pi^{2}$ and ^6^Σ^+^: $:1\sigma^{2},2\sigma^{2},3\sigma^{2},4\sigma^{2},5\sigma^{2},6\sigma^{2},1\pi^{2},7\sigma^{1},1\pi^{2}$ the dynamic correlation would be even smaller [1]. With a cc-pVQZ basis set and high correlation, the single reference CCSD (T) energy gap between the symmetric and asymmetric configurations is 136 cm^−1^ [23] which is reduced to 99 cm^−1^ in the RMR CCSD (T) method [14]. An additional extended discussion would be reported in a subsequent publication concerning strata/stratum (S/s) configuration.

We have reinvestigated the anion form at the QCISD (T), QCISD, CCSD (T), MP4SDQ, MP4D and full space CASSCF levels of theory employing Aug-CC-PVTZ and EPR-III basis sets. Although by our calculations the cyclic ${\text{ B}}_{2\;}N^{-}$ anion structure cannot be predicted to lie above the ${\tilde{X\;}}^{1}\;\Sigma_{g}^{+}$ ground state, it is notable that the lowest stable bent solution for B_2_$N^{\left(-\right)}$ should be a ^3^*B*_2_ state and not a single state.

Harmonic frequencies were determined at the QCISD/EPR-III//prop = EPR and characterized by 224.70 cm^−1^ ($\vartheta_{1}=\vartheta_{1}^{\prime },\;$ bending mode “$\pi_{u}$”) and 1203.42 cm^−1^ ($\vartheta_{2}$, symmetric stretching “$\sigma_{g}$”) and 1837.50 ($\vartheta_{3}$, asymmetric stretching “$\sigma_{u}$”). The IR and Raman intensities for $\vartheta_{3}$ are 1494.6 and 0.00, respectively, while the $\vartheta_{2}$” mode has intensity in Raman (39.97), but zero intensity in IR region. The valence orbital occupancy of ground state (${\tilde{X\;}}^{1}\;\Sigma_{g}^{+})$ is: $1\sigma_{g}^{2},1\sigma_{u}^{2},2\sigma_{g}^{2},3\sigma_{g}^{2},2\sigma_{u}^{2},1\pi_{u}^{4},4\sigma_{g}^{2},3\sigma_{u}^{2}$, while the lowest excited triplet state in the $D_{\infty h}$ representation for anion form has calculated (Table 1) and is predicted to be a ${\tilde{\;A}}^{3}\Pi_{u}$ state, lying 2.94 eV {${\tilde{\;A}}^{3}\Pi_{u}\left(a\right)-{\tilde{X}}^{1}\Sigma_{g}^{+}\left(a\right)$ in Table 1} above the ${\tilde{X\;}}^{1}\Sigma_{g}^{+}$ state (with the calculated value of 2.7 eV anion with photoelectron spectroscopy of B_2_N^(−)^).

The energies of |α〉 and |β〉 valance occupied MOs for the first triplet of exited state $\left(1\sigma_{g}^{2},1\sigma_{u}^{2},2\sigma_{g}^{2},3\sigma_{g}^{2},2\sigma_{u}^{2},4\sigma_{g}^{2},1\pi_{u}^{4},4\sigma_{u}^{1},1\pi_{g}^{1}\right)$ are $1\pi_{g}^{1}|\alpha \rangle =-0.03986$, $4\sigma_{u}^{1}|\alpha \rangle$ = −0.23701 and $\{1\pi_{u}^{2}|\alpha \rangle =-0.25466,\;-0.25136\}\;$ meanwhile, for |β〉 are $\left\{1\pi_{u}^{2}|\beta \rangle =-0.28802,-0.27730\;\right\}$, and $4\sigma_{g}^{2}|\beta \rangle =-0.01559$. The total energy for |α〉 is more than the total energy for |β〉 which indicates more correlation in anion form compared to the other two forms of B$N^{\left(+,\;0\right)}$.

However, the unrestricted QCISD (T) wave function is considerably spin-contaminated and characterized by a large *T*1 value. The ${\tilde{A}}^{3}\Sigma_{g}^{+}$ excited state corresponding to the promotion of an electron from the highest occupied molecular orbital (HOMO), $3\sigma_{u}$, to the $4\sigma_{u}$ MO is predicted to be considerably above the ${\tilde{\;A}}^{3}\Pi_{u}$ state.

4516 points have been used for fitting the atomic charges to electrostatic potential and the charges of ESP in this fitting are: {N = 0.891292, B = −0.945425, B = −0.945867}. Nitrogen is always positive while the two boron atoms are always negative and it is appeared conversely for the B_2_$N^{\left(+\right)}$ in which the nitrogen in cation is negative and two borons are positive in all ESP calculations. Both of the two highest occupied orbitals are predominantly nonbonding with the electron density localized mainly on the “terminal” boron atoms.

Isotropic Fermi contact coupling (IFCC) of B_2_N^(0)^ as a function of angles and distances is shown in Table 4 and Figure 3. Although the symmetry breaking cannot be seen in the IFCC{f(Δr)}, it can be seen in the IFCC{f(Δθ)} for the nitrogen in range of angles between Δθ= (50, −50) (Figure 3a'). On the other hand, the SB has not been observed for the Δ(Δθ) = $\Delta \theta_{B}-\Delta \theta_{N}$ in range of Δθ= (5, −5) (Figure 3a''). It seems that nitrogen in SB problem plays a major role and it depends on asymmetry bond changing and angle deformation interaction $\left(\pi_{u}+\sigma_{u}\right)$.

**Table 4:** **{**TQ^#^: Traceless Quadrupole moment (Debye-Ang)}; (**§**) Charges from ESP fitting and Isotropic Fermi Contact Couplings (MHz) (IFCC).

State	$E_{e}\left(Hartree\right)$	$B^{\delta q_{1}}–N^{\delta q_{2}}–B^{\delta q_{3}^{\left(§\right)}}$	$TQ_{xx}^{\#}$
(* N_e_)		IFCC(N,B_1_,B_2_)	$TQ_{yy}$
			$TQ_{zz}$
$\tilde{X\;}$ ^2^Σ^+^	$-104.0781959{\;}^{a}$	$\delta q_{1}=\delta q_{3}=-0.015{\;}^{a}$	$-1.8265{\;}^{a}$
$\left(C_{\infty v}\right)$	$-104.0820328{\;}^{a^{\prime }}$	$\delta q_{2}=0.03{\;}^{a}$	$0.8644{\;}^{a}$
(*17e)	$-104.0754917{\;}^{a^{''}}$	$-29.8,\;427.2,\;429.6^{\;a}$	$0.9621{\;}^{a}$
${\tilde{X\;}}^{2}\Sigma_{u}^{+}$	$-104.0781959\;{\;}^{b}$	$\delta q_{1}=\delta q_{3}=-0.015{\;}^{b}$	$0.9621{\;}^{b}$
$\left(D_{\infty h}\right)$		$\delta q_{2}=0.03{\;}^{b}$	$0.9621{\;}^{b}$
	$-104.159145{\;}^{f}$	$-29.9,\;428.2,\;428.2^{\;b}$	$-1.9241^{\;b}$
${\tilde{X\;}}^{2}\Sigma_{u}^{+}$	$-103.6396773^{\;d}$	$\delta q_{1}=\delta q_{3}=-0.0165{\;}^{d}$	$Q_{xx}=-16.5^{\;d}$
$\left(D_{\infty h}\right)$		$\delta q_{2}=0.033^{d}$	$Q_{yy}=-16.5{\;}^{d}$
(*17e)			$Q_{zz}=-19.8{\;}^{d}$
${\tilde{X\;}}^{2}\Sigma_{u}^{+}$	$-104.159145{\;}^{f}$	$\delta q_{1}=\delta q_{3}=-0.0045{\;}^{f}$	$0.8928{\;}^{f}$
$\left(D_{\infty h}\right)$	$-104.1355512^{\;n}$	$\delta q_{2}=0.009$	0.8928
			−1.7855
${\tilde{A\;}}^{2}$	$-104.1047582{\;}^{k}$	$137.9,\;817.0,\;817.0{\;}^{u}$	$-17.87{\;}^{u}$
(*17e)	$-104.0781729{\;}^{K^{\prime }}$		$-17.87{\;}^{u}$
			$-20.80{\;}^{u}$
${\tilde{\;B}}^{4}\prod_{g}$	$-104.0297022{\;}^{h}$	$20.26,\;377.84,\;377.84{\;}^{h}$	1.0334
(*17e)	$-104.0141196{\;}^{a}$		-2.4251
			1.3917
${\tilde{X\;}}^{1}$	$-104.1965676{\;}^{a}$	$\delta q_{1}=\delta q_{3}=-0.934$	$-14.8029{\;}^{a}$
(*18e)	$-104.2019114{\;}^{a^{\prime }}$	$\delta q_{2}=0.868{\;}^{a}$	$7.4015{\;}^{a}$
	$-104.1950169{\;}^{a^{''}}$	−14.98, 211.91, 211.91	$7.4015{\;}^{a}$
${\tilde{X\;}}^{1}$		$\delta q_{1}=\delta q_{3}=-0.934$	$7.4015{\;}^{b}$
(*18e)	$-104.1965676{\;}^{b}$	$\delta q_{2}=0.868^{\;b}$	$7.4015{\;}^{b}$
			$-14.803{\;}^{b}$
${\tilde{X\;}}^{1}$	$-104.1145486{\;}^{c}$	$\delta q_{1}=\delta q_{3}=-0.987$	$-15.7996{\;}^{c}$
(*18e)	$-104.1162881^{c^{\prime }}$	$\delta q_{2}=0.974{\;}^{c}$	$7.8992{\;}^{c}$
	$-104.112568{\;}^{c^{''}}$		$7.9004{\;}^{c}$
${\tilde{\;A}}^{3}\Pi_{u}$	$-104.0884685{\;}^{a}$	$\delta q_{1}=\delta q_{3}=-0.65{\;}^{h}$	$2.444{\;}^{h}$
(*18e)	$-104.1164696{\;}^{h}$	$\delta q_{2}=0.3$	6.5747
	$-104.0809447{\;}^{a^{\prime }}$		−9.0187
${\tilde{X\;}}^{1}$	$-103.7454365{\;}^{a}$	$\delta q_{1}=\delta q_{3}=0.805{\;}^{a}$	$8.0329^{\;a}$
(*16e)	$-103.8054934{\;}^{a^{\prime }}$	$\delta q_{2}=-0.611$	−4.0164
	$-103.7545006^{\;a^{\prime \prime }}$		−4.0164
${\tilde{X\;}}^{1}$	$-103.6023122{\;}^{m}$	$\delta q_{1}=\delta q_{3}=0.828{\;}^{f}$	$7.009{\;}^{m}$
(*16e)	$-103.3012055{\;}^{g}$	$\delta q_{2}=-0.656{\;}^{f}$	$-3.505{\;}^{m}$
	$-103.8377401{\;}^{f}$		$-3.505{\;}^{m}$
$\tilde{B\;}$ ^3^Σ_g_	$-103.7609202{\;}^{a}$	$\delta q_{1}=\delta q_{3}=0.763{\;}^{a}$	$-3.6992{\;}^{a}$
(*16e)	$-103.7767141{\;}^{h}$	$\delta q_{2}=-0.525{\;}^{a}\;$	−3.6992
	$-103.7628505{\;}^{a^{\prime }}$		7.3983

^(a)^ QCISD/EPR-III; ^(d)^ CASSCF(11,12)/UHF; ^(a^^')^ MP_4_D/EPR-III//QCISD/EPR-III; ^(m)^ CASSCF(10,12)/EPR-II; ^(a^^'')^ MP_4_SDQ/EPR-III//QCISD/EPR-III; ^(g)^ CASSCF(10,12)rohfAUG-cc-pvqz; ^(b)^ QCISD/EPR-III; ^(c)^ QCISD/EPR-II; ^(c^^')^ MP_4_D/EPR-II//QCISD/EPR-II; ^(c^^'')^ MP_4_SDQ/EPR-II//QCISD/EPR-II; ^(f)^ CBS-lq; ^(h)^ QCISD(T)/EPR-III; ^(n)^ CBS4O; ^(u)^ TD/EPR-II; ^(k)^ TD/EPR-III//QCISD(T)/EPR-III; ^(K^'^)^ TD/EPR-III//QCISD/EPR-III.

The role of nitrogen can also be discussed regarding the quadrupole moment. In Table 1, the traceless quadrupole moments in several ground and exited states are listed and it can be seen that the tensors of $TQ_{xx}$ and $TQ_{yy}$ are equal and positive, while $TQ_{zz}$ is negative when the symmetry of radical is $D_{\infty h}$ (in ground state). However, all three components are negative in the exited state for $\left(\;{\tilde{A}}^{2}\Sigma_{g}^{+}\;\right)$ and their values are in a large scale in comparison to those in ground state while for ${\tilde{\;B}}^{4}\prod_{g}$ they are irregular in the range of ground states. So, ${\tilde{B}}^{4}\prod_{g}-{\tilde{X\;}}^{2}$Σ+ interaction should be stronger than ${\tilde{A}}^{2}\Sigma_{g}^{+}-{\tilde{X\;}}^{2}\Sigma^{+}$. These results are the same for the anion in ground state of ${\tilde{\;A}}^{3}\Pi_{u}$ and the excited state of ${\tilde{\;A}}^{3}\Pi_{u}$ and the suitable interaction of ${\tilde{\;A}}^{3}\Pi_{u}-{\tilde{\;A}}^{3}\Pi_{u}$ can be predicted (it is ${\tilde{\text{X }}}^{1}\Sigma_{\text{g }}^{+}\;$ − $\tilde{\text{B }}$^3^Σ_g_ for cation). In other words, the quadrupole moment for BNB is highly sensitive to angle deformation and bond distance changing, so it can be discussed for any SBs in terms of quadrupole moment.

**Figure 4:**
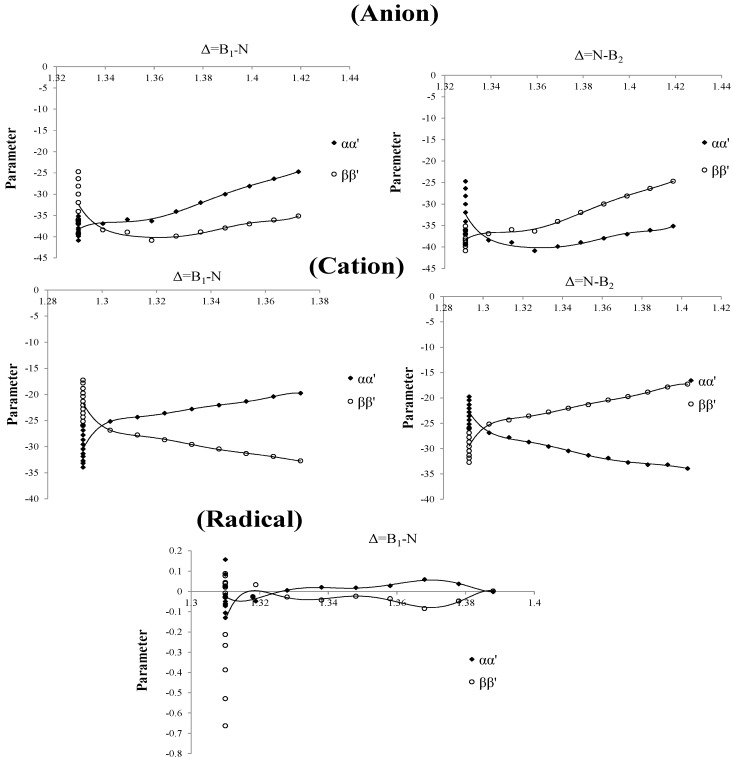
Parameters of Charge correction coefficients,* vs.* changing of boron and nitrogen distances for B_2_N^(−, 0, +)^ for anion , cation and radical.

Based on our previous work [59], we have modified the Columbic term of the Schrödinger equation for the definition of parameters $“\alpha \alpha^{\prime }\text{ and }\beta \beta^{\prime }”$. The left part in the chart for the three curves of anion, cation and radical of these parameters are straight towards up and down (Figure 4) and their values are 1.293, 1.310 and 1.329 for cation, radical and anion, respectively. In the right part there is a point splitting between two curves of $\alpha^{\prime }$ and $\beta^{\prime }$(1.303, 1.316 and 1.339 for cation, radical and anion, respectively) indicating a proper region for charge stability distribution in the SB problem.

## 5. Conclusions

In this study, we have shown that the SB problem is not a real phenomenon; it is a hidden function depending on various variables such as charge distribution, bond length, IFCC, primitive Gaussians, trial wave-function properties, frozen core electrons and most importantly, non-Born–Oppenheimer approximation approach. While the nuclear repulsion energy of B_2_N^(−, 0, +)^ in Born–Oppenheimer approximation mostly depends on the variables such as B-N bond length, using large and larger basis sets and more and more electron correlation are doomed to result in wrong limit for the energy level of SB barriers or SB estimation. It is prudent to employ a highly correlated method which can use a large number of reference determinants to recover dynamic and static correlations. We have shown that the SB is generally applied to the failure of the electronic wave function in order to be transformed as an irreducible representation of the molecular point group, so the failure of the electronic wave function is purely artifactual. It is not wise to conclude that the only special level of theory on the symmetry breaking for BNB is real (which has been concluded in reference [9]) because there exist some hidden variables in the electronic wave functions which should be considered. We have found that the symmetry breaking (SB) for some hidden variables (such as charge distribution) not only exhibit an energy barrier, it also creates several SBs through the asymmetry stretching with bending mode interaction.
